# Predicting Postoperative Complications in Cancer Patients: A Survey Bridging Classical and Machine Learning Contributions to Postsurgical Risk Analysis

**DOI:** 10.3390/cancers13133217

**Published:** 2021-06-28

**Authors:** Daniel M. Gonçalves, Rui Henriques, Rafael S. Costa

**Affiliations:** 1 IDMEC, Instituto Superior Técnico, Universidade de Lisboa, Av. Rovisco Pais 1, 1049-001 Lisboa, Portugal; dmateusgoncalves@tecnico.ulisboa.pt (D.M.G.); rafael.s.costa@tecnico.ulisboa.pt (R.S.C.); 2 INESC-ID, Lisboa Portugal and Instituto Superior Técnico, Universidade de Lisboa, R. Alves Redol 9, 1000-029 Lisboa, Portugal; 3 LAQV-REQUIMTE, NOVA School of Science and Technology, Campus Caparica, Universidade NOVA de Lisboa, 2829-516 Caparica, Portugal

**Keywords:** postsurgical risk, cancer, machine learning, survey, clinical prognosis, postoperative outcomes

## Abstract

**Simple Summary:**

Structured survey on the predictive analysis of postoperative complications in oncology, bridging classic risk scores with machine learning advances, and further establishing principles to guide the design of cohort studies and the predictive modeling of postsurgical risks.

**Abstract:**

Postoperative complications can impose a significant burden, increasing morbidity, mortality, and the in-hospital length of stay. Today, the number of studies available on the prognostication of postsurgical complications in cancer patients is growing and has already created a considerable set of dispersed contributions. This work provides a comprehensive survey on postoperative risk analysis, integrating principles from classic risk scores and machine-learning approaches within a coherent frame. A qualitative comparison is offered, taking into consideration the available cohort data and the targeted postsurgical outcomes of morbidity (such as the occurrence, nature or severity of postsurgical complications and hospitalization needs) and mortality. This work further establishes a taxonomy to assess the adequacy of cohort studies and guide the development and assessment of new learning approaches for the study and prediction of postoperative complications.

## 1. Introduction

Cancer is among the leading causes of death of the 21st century. In the United States, as of 2020, the number of new cases of cancer was estimated to surpass 1,800,000 and deaths due to cancer were close to 600,000. The survival rate within 5 years for these patients is currently around 65% [1]. The morbidity and mortality associated with cancer can result from direct consequences of the disease but can also occur due to operative and postoperative complications [2,3], generally lowering the survival rate and, in certain types of cancer, aggravating the recurrence rate [4].

The health impact of cancer surgeries is difficult to predict, due to the high number of factors pertaining to the physiological resilience of an individual, the cancer profile and the nature of the undertaken surgeries. Considerable scientific efforts have focused on postoperative complication risk assessment tools for cancer and general surgeries. These tools generally aim at anticipating mortality and morbidity risks in order to guide surgical design and care decisions [5]. With advances on the technology and health data analysis, an increasing amount of studies identify the main factors propelling postoperative complications and, considering these factors, propose new risk tools, or recalibrate existing ones [5]. In this context, medical professionals are assisted when deciding whether a surgery is viable for a patient, while patients can more easily manage expectations associated with potentially high-risk surgeries. From a clinical perspective, the risk scores are also determinant in choosing the course of actions, such as additional testing, specific prehabilitation programs, or supportive measures that should be followed during the intraoperative or postoperative periods [5].

**Figure 1:**
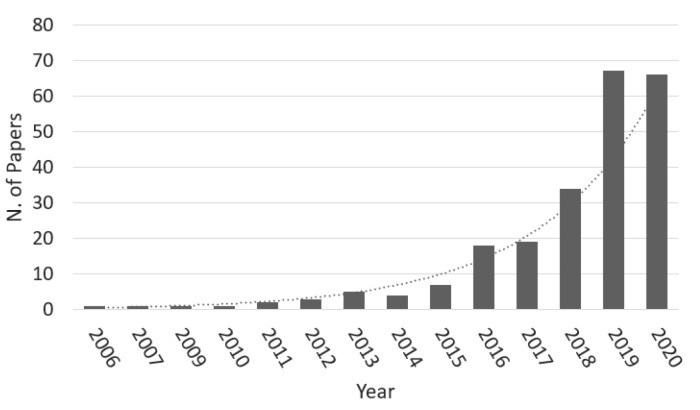
Number of publications per year: search results for the joint keywords “machine learning” and “cancer surgical risk” on PubMed, accessed on January 2021.

Recent advances in machine learning (ML) techniques and the increasingly large cohort of studies are radically changing cancer surgery prognostication. The number of papers related to this matter has steadily increased, as shown in Figure 1. Although the results achieved by ML models are generally comparable or better than classic models (e.g., Cruz and Wishart [6]), there is considerable agreement that there is still space for further improvements once more extensive data sets become available [7]. In fact, ML has been explored to predict cancer-related outcomes for over 30 years [8,9].

This work proposes a structured survey on the problem of predicting postoperative complications in the oncological domain. To this end, we first establish a taxonomy to guide the design of cohort studies and the development and assessment of new learning approaches for predicting postoperative complications. Contextualized by this taxonomy, this paper then provides a comprehensive survey of classical approaches (risk calculators, scores and indexes) and ML advances for postsurgical risk analysis. Most of the ML-based works we discuss were published in the last 2–6 years.

The paper is structured as follows. Section 2 offers essential background on classic and ML-based stances to prognostication. Section 3 introduces the proposed taxonomy. Section 4 overviews traditional and state-of-the-art approaches for postsurgical risk analysis against the introduced taxonomical dimensions. Section 5 discusses the findings, offering further insights to address the target problem. Finally, concluding remarks and implications are synthesized.

## 2. Background

### 2.1. Classic Prognostication: Calculators, Scores and Indexes

Efforts to predict postoperative complications have been a constant since the dawn of medical practice, progressively using more sophisticated means and consequently obtaining more accurate results [10]. Medicine transitioned from intuitive guessing based on expertise knowledge into having indexes, scores and calculators to aid the medical assessment of preoperative and postoperative patient states. Solutions such as the original American Society of Anesthesiologists (ASA) score for the classification of physical status (PS) [10] started to emerge in the past century as an attempt to standardize “Operative Risk”. The ASA-PS scale still provides to this day satisfactory results in predicting postoperative complications and death [11]. However, this classification is inferred from input variables that inherently depend on the subjective perception of the medical professionals [12]. ASA-PS is a point system with various parameters whose evaluation is not standardized, thus is associated with high variability among similar users. Despite these criticisms, ASA-PS is still used today since studies suggest that its result roughly transduce the risk of morbidity and mortality [11]. In fact, it has laid the foundation for other classification systems.

A considerable amount of calculators based on statistical methods, such as multivariate regression or correlation analysis, have been proposed since then, including those proposed by Copeland et al. [13] (POSSUM) or Bilimoria et al. [14] (ACS). Although generally more expressive, they are susceptible to generalization problems and commonly either assume independence between the monitored variables or simplistic linear combinations [6].

To obtain more complete, accurate and detailed postsurgical information, hospitals often make use of more than one of these classic systems in order to dilute errors among the voting of all the predictors. In addition, distinct predictors can provide complementary views of either the same or different postoperative outcomes. The typical outcomes associated with cancer prognostication tools are as follows: (1) risk of postoperative morbidity (presence, nature and severity) and mortality; and (2) cancer recurrence probability. In what strictly concerns postoperative complications’ risk, recurrence is commonly left out of the equation, although it can be conceptually contained in the larger problem of prognostication [15,16]. The referred outcomes are usually tied to specific time horizons, specific complications and/or specific demographics.

Typically, classic prognostication tools use a limited set of variables that can easily be monitored or statistically inferred by physicians. With the rise of clinical big data [17], cohorts are increasingly larger and new variables pertaining to the clinical, molecular, demographic and the exposomic profiles of patients are now available. Today “high-throughput diagnostics” mean that decisions are made from high-dimensional data spaces, paving new opportunities for clinical predictive models in the precision medicine era [18].

### 2.2. Machine Learning Models

Although classic prognostication systems are still widely used within hospitals to predict postsurgical risks, more advanced approaches have been proposed in the last decades to address the aforementioned challenges faced by classic approaches [19]. ML approaches outperform classic methods when the target predictive task is fundamentally non-linear, being able to learn models from multicolinear variables with complex interdependencies. Although ML is based on statistics and probability, the differentiating characteristic is the ability of ML approaches to make inferences or decisions beyond the capabilities of conventional statistics. The problem of learning from data and generalizing to inference can be done in either a supervised or unsupervised setting [20]. In supervised learning, the individuals from a given cohort study are known to have well-defined postoperative outcomes (whether categorical or numeric), and the goal is to learn a mapping function—the predictive model—between historical data and the postsurgical outcome. In unsupervised settings, the goal is rather learning relevant associations from the available cohort data, including the discovery of temporal patterns of recovery progression, the learning of generative models of postsurgical health-and-care outcomes, or the clustering of individuals into risk groups [21]. This review focuses on supervised learning (predictive approaches).

What follows is a brief description of the principles underlying classic ML approaches for predictive tasks. The listed models were chosen due to their inherent simplicity, popularity and proved usefulness in the clinical domain. They set the generic foundations for more advanced variants that are discussed more thoroughly in Section 4. The k-nearest neighbors(kNN) algorithm, one of the oldest and simplest ML methods [22], identifies the most similar individuals (the neighborhood) to the individual under assessment for either classifying outcomes or estimating risk scales. On the side of probabilistic approaches, naïve Bayes (NB) [23] assumes conditional independence among the input variables to calculate outcome-conditional probabilities against the fitted distributions per variable. Decision trees (DT) [24], abundantly used in clinical predictive settings (with both categorical and numeric outcomes), given their inherent simplicity and interpretability, focus on local discriminative patterns through the use of information theoretic measures. Tree ensembles, obtained through the use of bagging and bootstrapping principles [25], can be considered to minimize the underfitting propensity of a single decision tree. Random forests (RF) and extreme gradient boosting (XGBoost) are two paradigmatic cases. Artificial neural networks (ANN) offer the possibility to learn non-linear mappings, using brain-inspired pathway connections organized in well-defined layers [26]. In the last years, motivated by the evolution of technology and size of cohort studies, deep neural networks have been popularized, due to their capacity to model complex problems. Support vector machines (SVM) [27] aim at identifying hyperplanes able to either optimally separate individuals with different outcomes (classification) or approximate quantities with minimum errors (regression). To learn non-linear surfaces, kernels—transformations of the feature space—are considered. SVMs are still not as widespread for cancer prognostication as classic regression models or neural networks [6]. A comprehensive discussion on the potentialities and limitations of these approaches and other variants for postsurgical risk analysis is provided along Section 5.

## 3. Taxonomy of Postsurgical Risk Analysis

**Figure 2:**
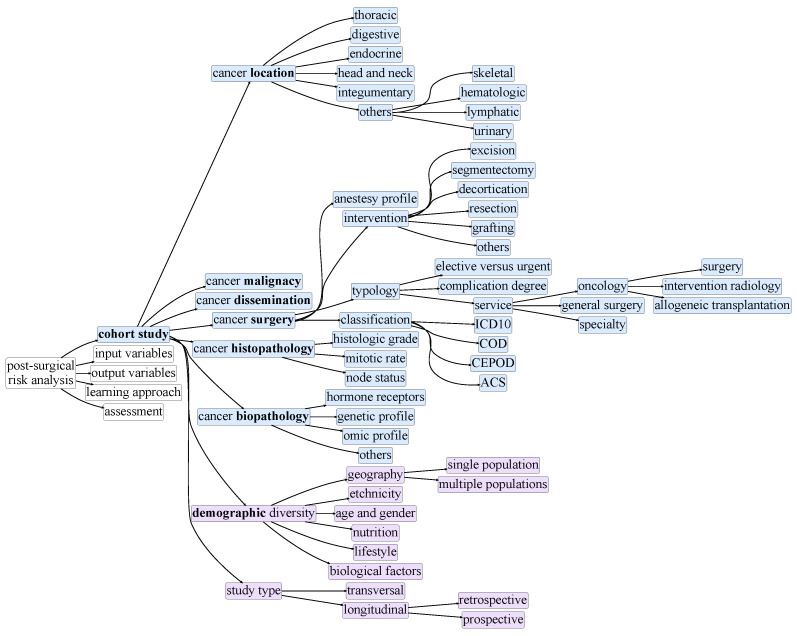
Taxonomy for postsurgical risk analysis: cohort study.

**Figure 3:**
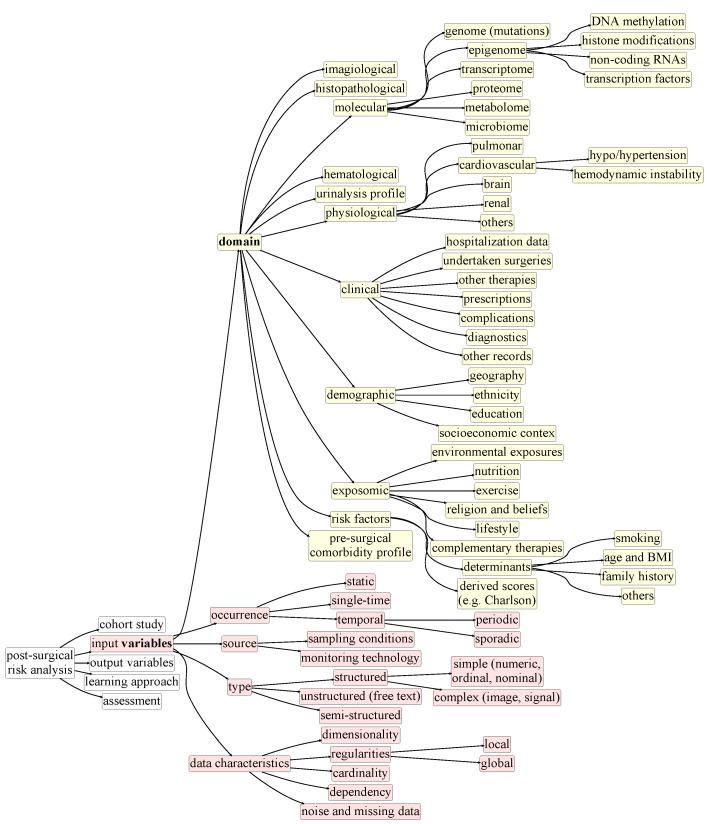
Taxonomy for postsurgical risk analysis: available data (*input*).

**Figure 4:**
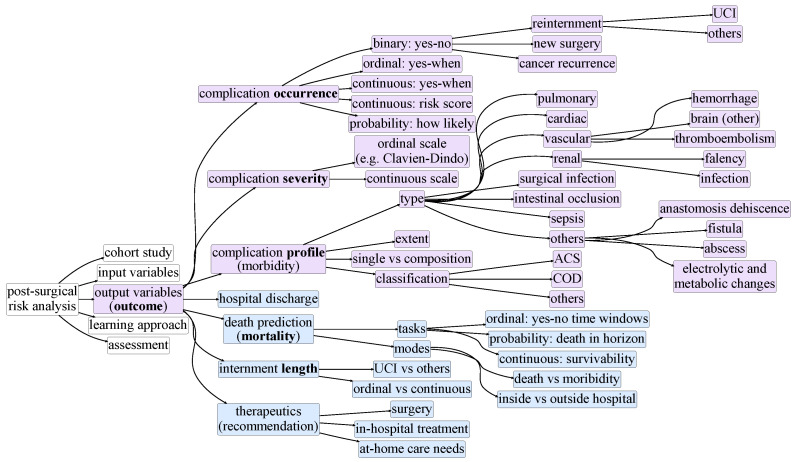
Taxonomy for postsurgical risk analysis: postsurgical outcomes (*output*).

**Figure 5:**
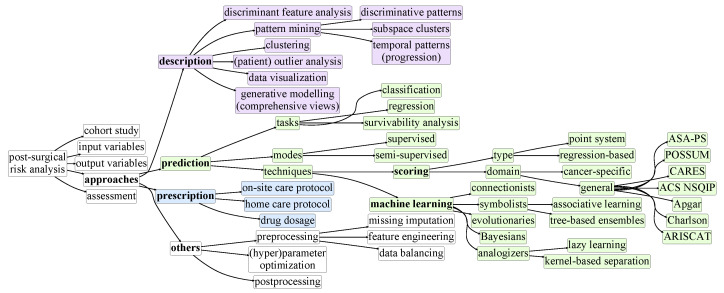
Taxonomy for postsurgical risk analysis: descriptive, predictive and prescriptive approaches (*input–output mapping*).

**Figure 6:**
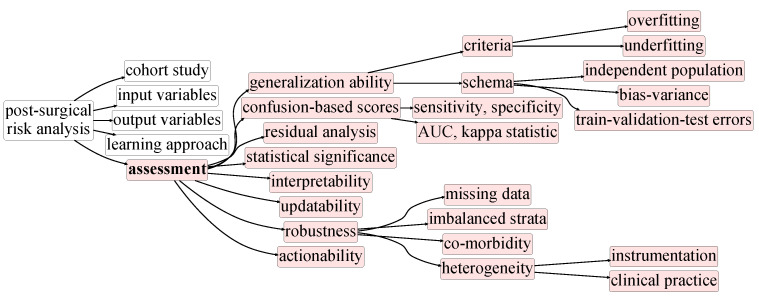
Taxonomy for postsurgical risk analysis: assessment.

Figure 2, Figure 3, Figure 4, Figure 5 and Figure 6 compose a taxonomy to offer a structured understanding of the diverse aspects associated with the postsurgical risk analysis from cohort studies. This taxonomy is further presented as a roadmap to (1) guide the design of cohort studies in terms of their scope (Figure 2), input data (Figure 3), and study outcome (Figure 4), (2) guide the development and assessment of approaches (Figure 5 and Figure 6) to study and predict postoperative complications, and (3) improve the data-quality collected in multicenters.

Identifying the characteristics of the target cohort study (Figure 2) is an essential step toward the assessment of postoperative complications, whether the cohort study is at the design stage or already in place. Important aspects include the following: (1) cohort size; (2) the demographic diversity of the target population—geography, ethnicity, age, gender, education, or lifestyle of the individuals; (3) the presence of multiple hospitals or care facilities with possibly different practices, instrumentation or standards for data collection; (4) whether the target population is homogeneous or heterogeneous with regard to cancer location—including thoracic (breast, lung), digestive (colorectal, stomach, intestine), endocrine (prostate, ovary, thyroid), brain, skin, skeletal, hematologic, lymphatic, and urinary (bladder, kidney); (5) the diversity of the population regarding cancer malignancy, dissemination, and other histopathological and biological features, as well as the characteristics of the undertaken surgical interventions (Figure 2); and (6) the extent and recurrence of patient health-and-care monitoring during pre- and postsurgical stages. All these variables are essential to determine the (i) ability to conduct sound statistical assessments, (ii) generalization ability of the target learning approaches, and (iii) coverage and external applicability of the target predictive models.

The available cohort data also determine the nature of the postsurgical analysis (Figure 3), whether it is primarily driven by the undertaken surgery (procedure and outcomes) and risk factors of the patients, or further able to integrate additional sources of information, including the following: (1) cancer histopathological features (such as histologic grade and mitotic rate); (2) molecular measurements (genetic mutation profile, epigenetic profile, gene expression, the concentration of specific non-receptor proteins and metabolites of interest, glycosylation and other molecular additions on key proteins); (3) hospitalization data (including details on the observed complications, applied prescriptions, and undertaken therapies); (4) exposomic data (including the nutrition, exercise and lifestyle profile of the patients); (5) hematologic and urinalysis data; and, among others, (6) at-home care data. The nature of the monitored variables—domain (whether they are numeric, ordinal, nominal, imagiological, temporal, or semi-structured) and characteristics (distribution, susceptibility to noise and missing values)—determine the ability to learn comprehensive models of postsurgical health progression.

A secondary essential step is to identify the target postsurgical outcomes (Figure 4), as the focus can be placed on different ends, including determining (1) the occurrence and severity of postsurgical complications, (2) the nature of complications (morbidity), (3) the need for rehospitalization or new surgeries, (4) the cancer recurrence, (5) the internment length, or (6) the survivability of the individuals (mortality). The selection of the outcomes determine whether the learning task can be better formulated as a classification, regression, unsupervised, or survivability problem. Illustrating that, considering the assessment of the postsurgical occurrence of complications, it can be formulated as a yes–no classification problem (where the yes can be further refined into time horizons) or as a yes–when regression problem. Outcomes based on severity indexes typically rely on ordinal scales, such as Clavien–Dindo [28], while outcomes grounded on the assessment of the complication can rely on classification standards, such as ACS [14]. In this latter case, determining the granularity at which complications can be predicted is an important step and should guarantee the presence of a representative number of cases per complication. Common classifications typically include cardiovascular, pulmonary, renal, and surgical-specific (localized infections, fistulas, abscesses) complications. Figure 4 provides further taxonomic details on possible outcomes for postsurgical risk studies.

Once cohort data are available (input) and the desirable postoperative outcomes are fixed (output), input–output mappings can be established using one of three major approaches: descriptive, predictive and prescriptive approaches (Figure 5). Descriptive approaches can be applied to different ends: (1) discovery of discriminative patterns of postsurgical risk, and temporal patterns of recovery progression; (2) learning generative models able to comprehensively capture postsurgical health-and-care outcomes; (3) discriminant feature analysis; (4) clustering of individuals into risk groups; (5) visual analytics to support the study of correlations; and (6) analysis of outlier individuals, including individuals with comorbidities or unexpected outcomes. One example of mining pre-surgical patterns to discriminate postsurgical outcomes in the oncological context is given in [29]. In contrast with descriptive approaches, predictive approaches produce models that can be readily applicable on new patients to assess their postsurgical risks. Predictive approaches, the focus of this manuscript, can further benefit from semi-supervised learning principles when not all information regarding the postsurgical patient outcome is available. Finally, the previous approaches can be complemented with optimization and simulation studies (prescriptive setting) in order to plan on-site and at-home care protocols.

The resulting descriptive, predictive and prescriptive models should be subjected to careful assessment (Figure 6) to guarantee their generalization ability, outcome sensitivity, statistical significance, completeness, interpretability, updatability for ongoing cohort studies, and actionability.

## 4. Postoperative Prognostics: A Literature Review

Prognostication tools are in a state of constant improvement. The first formal studies date back to the 1940s [10]. Out of the diversity of outcomes introduced in Figure 4, the survey primarily focuses on two major predictive ends, morbidity and mortality, strongly correlated and denotative of postoperative complications.

**Methods.** For this survey, studies on cancer surgery, ranging from traditional statistics to modern machine learning models (in accordance with predictive taxonomic associations in Figure 5), were analyzed. The search strategy for peer-reviewed manuscripts was performed through Google Scholar and PubMed engines under the following term-sets: “cancer postoperative complications”, “cancer prognostic”, “postsurgical complications” or “surgery prognostic”, coupled with “prediction” or “machine learning”. No filters were added to the search queries since the objective was to obtain the entire spectrum of publications across a vast time frame. This search process was conducted in January 2021. The results of each search were very extensive, ranging from twenty five thousand to more than one million hits, depending on the search query. The title, abstract and keywords of each publication were firstly analyzed to filter irrelevant manuscripts. The ones deemed relevant are here described.

### 4.1. Traditional Risk Scores Studies

Classical statistical studies on postoperative complications made their way into clinical use and were adopted by hospitals to support medical decisions for nearly one century [10]. Most of these clinically adopted scores, indexes and calculators are based on simple statistical methods, which are largely considered reliable and less susceptible to the same degree of distrust that some machine-learning methods still face today, due to unfamiliarity and the “black-box” character typically attributed to them. Table 1 lists the major traditional statistical studies for postoperative prognostics.

**Cohort–outcome relationship.** As highlighted in the input and output taxonomic dimensions in Figure 2, Figure 3 and Figure 4, the characteristics of the monitored population are a determinant factor. The POSSUM score was created to predict the mortality risk from a general surgery cohort [13]. Although it has wide applicability, POSSUM discards the oncology-specific context. In the same line of thought, CARES surgical risk calculator was developed from a cohort of individuals undertaking cardiac surgeries [35]. Being more specific than POSSUM, CARES predictions for in-hospital death and morbidity are also more adequate for application in patients submitted to cardiac interventions. Although extrapolation is possible, further testing of the gathered scores is advised, as generalizing predictions for other clinical specialties is generally susceptible to errors.

There are studies which rely on massive cross-hospital populations comprising millions of individuals, such as the ACS NSQIP, which makes use of data collected from 393 American hospitals, amounting to nearly 1,500,000 patients [14]. Studies with such extensive data sets are able not only to yield better predictions, but accommodate less-trivial outcomes other than mortality and morbidity targets. ACS offers 8 outcomes: two “primary” scores dedicated to mortality and morbidity, and 6 “secondary” scores dedicated to classes of complications (Figure 4). Each score is predicted by its own regression model.

Specialty-specific scores often rely on considerably small cohorts with a few hundred individuals. The Surgical Apgar Score considered only 303 patients for training the statistical model [39]. As only three variables are collected to make the predictions (ratio of 100 records per variable), the statistical significance of the inferred associations can be assessed and further validated in validation sets.

These observations show that the nature of the surgical cohort available at the time of research and development is a crucial factor that can limit the final outcome. Larger populations and broader demographics, in accordance with the dimensions introduced in Figure 2, contribute to wider applicability and greater diversity of context-specific outcomes. As highlighted in Figure 3, the size of the cohort, as well as its dimensionality, sparsity, regularities and dependencies among the collected variables are determinant factors across classic point systems [43,44].

The monitored variables throughout the majority of the reviewed traditional statistical studies are generally limited to clinical, clinicopathological and hematological variables. Very seldom did the studies include demographic, socioeconomic, exposomic and a more comprehensive molecular profile of the individuals (as surveyed in Figure 3), important variables that could promote the international applicability of each study. One case is the ACS NSQIP Surgical Risk Calculator [14], which accounts for demographic data collected from over 393 American hospitals, thereby having a solid and proved national applicability.

**Point systems.** The novelty behind each one of the reviewed scores (Table 1) is generally attributed to the type of model used, cohort extent, or the nature of the monitored variables considered to train the model. There are models ranging from simple scoring point systems to regression models. The Charlson Comorbidity Index (Charlson et al. [31]) or the Surgical Apgar Score (Gawande et al. [39]), used to classify disease severity and also predict in-hospital death, are good examples of point systems that sum the results or apply a simple statistic, using the devised points. These methods generally lack the generalization guarantees, adaptability and complex modeling capabilities that ML models easily attain nowadays. Instead, point systems can be manually tuned, based on a number of factors previously studied and proven to have impact on a certain outcome.

**Logistic regression.** Alternative risk scores make use of more advanced training or more complex models to make their prediction. In fact, this is the case with the majority of the reviewed scores in Table 1. The difference between regression and point systems or weighted indexes is small in practice and resides solely on the way in which the weights of each factor are approximated from the available cohort data. Multivariate logistic regression is the most widely used model, generally employed when the target outcome is of a binary nature and essentially obtained by minimizing the loss between the actual outcomes and the sigmoid of the computed scores produced by linear regression [21].

### 4.2. Machine Learning Studies

More recently, ML also stepped into the field of postoperative prognostication, with the number of yearly contributions considerably rising in last years as shown in Figure 1. A comprehensive list of machine-learning studies for postsurgical risk analysis is presented in Table 2.

**From traditional statistics to machine learning.** The median publication year of the classical postsurgical studies corresponds to 2001, while ML studies correspond to 2015. Along these fourteen years, the computational resources and techniques evolved, as well as the size and characteristics of the conducted cohort studies. When comparing Table 1 and Table 2 in light of the introduced taxonomy (Section 3), differences are notorious, particularly differences pertaining to the nature and extent of the monitored variables. More recent ML models make use of genomic, biophysiological, radiomic, demographic and socio-economic variables. By these means, studies employing ML models dispose of a broader individual’s profile to foster prediction capabilities, as well as assess their adaptability and reusability across different clinical and surgical areas (Figure 6). Another characteristic differentiating ML from classical studies is the discrepancy of their clinical translation footprint. Many classical statistical studies were driven by (or in strong collaboration with) medical professionals and, despite their inherent simplicity and generalization difficulties, are widely adopted. ML approaches are commonly more experimental in nature and generally show limited cross-hospital applicability.

**Machine-learning contributions.***Naïve Bayes* (NB), commonly chosen when variable dependencies are not determinant, was selected in four of the ML prognostication studies in review [48,49,50,51]. Due to its outcome-conditional behavior, NB did not score as the best method across all of these four studies. According to Danjuma [48], its simplistic nature is capable of improved prognostics when compared with logistic regression, and Parmar et al. [49] shows to be competitive with SVMs, NNs and RFs.

The *k-nearest neighbors* (kNN) algorithm, one of the most intuitive and simple methods available, partakes in the Wang et al. [50] prognostication study of post-cystectomy mortality. The authors considered the application of the Euclidean distance to measure individual similarities from nominal and ordinal data, a disputable choice, given the categorical nature of variables. The size of the neighborhood, *k*, was shown to be determinant to avoid the impact of outlier individual profiles (*k* too low) and non-local dominance (*k* too high). Despite the inherent merits of kNN, it was shown to not be competitive with other ML peers for the considered prognostic outcomes.

*Decision trees* (DTs), non-parametric models able to capture non-linear yet simplistic discriminative associations between variables and outcomes, are popular in prognostication, due to their high interpretability and suitablity for mixed variable domains, numerical and categorical. Danjuma [48] used DTs to predict mortality within 1 year from surgery. The results shown their efficacy for the targeted ends, with the efficacy only slightly surpassed by artificial neural networks. Fuzzy DTs are similar to classic DTs, with one difference residing on the explicit accommodation of class-conditional strengths along paths, instead of crisp classification. Khan et al. [45] applied both fuzzy and crisp DTs for breast cancer survivability and showed that, despite the absence of statistically significant differences in performance, fuzzy logic brings broader insight to the predictions, further promoting the interpretability of postsurgical models.

*Support vector machines* (SVMs), popular choices although not as interpretable for healthcare practitioners as DTs or kNN, were considered in four of the studies in our review. Chang et al. [46] showed that a linear kernel SVM for predicting 3-year mortality, although yielding comparable performance with logistic regression, was not ranked among the best ML models since the collected survivability-conditional data are hardly linearly separable. Soguero-Ruiz et al. [52] tested linear and non-linear kernel SVMs over well-diversified sets of variables extracted from clinical records’ free-text, hematological exams and vital signs. Non-linear kernels generally yielded better results, especially when heterogeneous types of variables were considered. In contrast with some of the previous findings, Thottakkara et al. [51] highlight the good performance of linear SVMs, showing that the nature of the undertaken cohort study and target outcome are determinant. Lastly, polynomial kernel SVMs were further assessed by Wang et al. [50] to predict 5-year mortality, yielding accurate results, yet not showing competitive sensitivity levels.

*Neural networks* (NNs) are used in five of the reviewed studies. Kim et al. [53] used DeepSurv, a class of deep feed forward neural networks to make predictions about survivability. The structure and hyperparameters of DeepSurv models were subjected to grid search optimization. This study shows DeepSurv to be the best model, surpassing RFs and traditional survivability models, such as Cox proportional-hazards. Allied with various feature selection methods, Parmar et al. [49] tried to predict 3-year mortality on a high-dimensional data set with 101 patients and 404 features. After feature selection, only 30 features remained, and out of all the models, NNs yielded superior AUC and stability across the tested settings. Danjuma [48] showed that feed forward NNs can outperform DT and NB to predict postoperative life expectancy in lung cancer patients.

Chang et al. [46] considered multi-layered feed forward neural networks, trained using the Levenberg–Marquardt algorithm, as well as fuzzy network referred to as an adaptive neuro-fuzzy inference system (ANFIS) based on rules generated from output membership functions. Among the assessed ML models, ANFIS was found to be the overall best method, further contrasting the poor performance of simplistic feed forward NNs. Lastly, Wang et al. [50] also compared various NNs in their set of ML models. Architectural decisions and hyperparameters were subjected to optimization to guarantee the generalization ability of the models. In addition to classic NNs, Wang et al. [50] further assessed extreme learning machines (ELM). A key feature of ELM is that the weights and bias between the input and the hidden layers are randomly assigned, whereas the weights between the hidden and the output layers are analytically determined using the Moore–Penrose generalized inverse operation. The authors concluded that a regularized version of ELM, RELM, yields the best generalization followed by ELM, while simpler multi-layer perceptrons are less competitive, yielding a performance comparable with kNN.

*Ensemble learning*, aiming at reducing the sources of noise, bias and variance by combining multiple ML models, are considered in three of the reviewed studies. Parmar et al. [49] assessed the role of random forests (RF) as prognostic biomarkers of head and neck cancer. The results suggested that RFs yield competitive performance and stability across testing partitions. Zięba et al. [47] proposed a boosted SVM model to solve inner- and between-class imbalanced data problems. The problem of uneven data is solved by proposing weighted error function with different misclassification costs for positive and negative examples, respectively. The boosting algorithm used is AdaBoost, which makes use of weak learners (in this case SVMs) to iteratively adjust the data weights in order to increase the significance of misclassified weights, tackling outcome imbalance. The results revealed good performance and proved the ability to overcome imbalance-induced bias. Parikh et al. [54] used RFs and gradient boosting (GB), both tree based ensemble models. RFs and GB were tuned, using grid search to optimize the number of tree estimators, tree depth-related parameters, and, in particular for GB, the loss function and learning rate. Gradient boosting behavior is analogous to AdaBoost, with the difference residing on the assessment of weak learners—while AdaBoost weights data points, GB adapts gradients in the loss function. Both models showed superior results with a positive predictive value superior to that of traditional statistical values. They also helped in recognizing less-trivial relevant predictive variables [54] for which the domains are listed in Figure 3, previously ignored by traditional statistical methods.

### 4.3. Preprocessing

Preprocessing the available cohort data is generally entailed to support the subsequent learning. This quality leveraging process is inherent to every surveyed study, yet sparingly documented. Out of the analyzed publications, only 38% actually disclosed the undertaken processing strategies. Understandably, preprocessing needs depend on the unique aspects of each cohort study (Figure 2, Figure 3 and Figure 4). For instance, the limited number of individuals and high number of monitored variables in some cohort studies creates generalization difficulties—described as “the curse of dimensionality” [55]. Different preprocessing principles are employed by statistical and ML studies to handle this problem in accordance with its severity, outcome-conditional data regularities, and the behavior of the applied methods. What follows is a brief description of the major preprocessing challenges, together with the principled solutions found among the reviewed literature.

In postsurgical cohort studies, *missing values* are a common result of unavailable data at the time of registry, exam dispense, unchanged records since last examination, or a product of human error, among other sources [56]. Since a considerable portion of the surveyed ML models cannot handle missing values, record removals and missing value estimation are commonly pursued [56]. Given the limited size and dimensionality of most cohort studies, the removal of patients or variables with missing entries cannot be afforded, and imputation using mean, median, mode or dedicated missing labels are a commonly preferred option [51,57]. Another solution consists of using methods that are well-prepared to handle missing values, including logistic regressors, NBs, DTs and NNs [58]. Alternative distance-based methods, such as kNN, can take into account missing occurrences when comparing patients, bypassing biases caused by imputation techniques [58].

*Outcome imbalance* is commonly present in postoperative predictive problems [47]. Due to this inevitable fact, depending on the model used, predictions can be biased toward the majority class. This is particularly problematic when the minority class represents negative effects, such as death, or a morbidity factor, such as a particular postoperative complication [59]. Undersampling and oversampling are commonly pursued resampling options [59], yet respectively challenged by information loss and synthetic-duplicate biases. Combining such options can be alternatively considered [60]. To tackle the limitations of resampling options, outcome imbalance can also be addressed out of the preprocessing stage by selecting models sensitive to the effects of imbalanced data. As previously introduced, Zięba et al. [47] introduced SVM-based ensemble principles that were proved to be efficient at dealing with data imbalances.

*High dimensionality* further poses generalization challenges, predisposing overfitting risks for small cohort studies. Several studies alleviated the learning stage by pursuing feature selection as a preprocessing step, such as Chang et al. [46], Parmar et al. [49] or Parikh et al. [54], to improve model interpretation, efficiency, and generalization ability by reducing model variance. Complementarily, feature extraction techniques able to capture dependencies between features, such as forms of principal and discriminant component analysis [61,62], were also pursued in prognostication studies [51]. A recent study showed that issues pertaining to assessment and feature selection choices are commonly associated with optimistic results [63]. This observation should not be neglected upon consulting the surveyed works, given the general scarcity of reproducible information regarding the entailed preprocessing steps.

### 4.4. Prognostic Accuracy

Assessing postsurgical prognostication models should, beyond accuracy-loss views, further account for the generalization, and interpretable, updatable and actionable capacities of the models, as introduced in Figure 6, to guarantee their clinical translation. The majority of the reviewed publications with *categorical outcomes* rely on confusion-based predictive metrics. Confusion matrices trace actual versus predicted outcomes, enabling the extraction of various metrics, including sensitivity (also referred to as recall or true positive rate), specificity (false positive rate), accuracy, precision and *F*-measures. The former three being common choices for balanced outcome settings when the false positive rate is significant, while sensitivity and the latter two are the common options for imbalanced settings or when the focus should be majorly placed on a specific outcome of interest [64]. The receiver operating characteristic (ROC) curve, and the corresponding area under the curve (AUC), are more comprehensive measures of outcome separability. Considering non-binary categorical outcomes, ROC curves can be inferred per outcome and their joint AUCs (or overlap ROC plotting) used to assess the separation ability. Bridging back to the reviewed works, only three neglect ROC-based assessments [31,33,39].

Despite the relevance of the previous evaluation criteria, they are unable to assess the statistical significance of the outcome predictions. To this end, the Pearson’s Chi Square Test can be applied to ensure if prognostics arose by chance [65]. In total, eight of the reviewed studies conducted this statistical test [13,30,31,32,33,35,39,40]. Much like the coefficient of determination, the Hosmer–Lemeshow Test (HL) is a measure of the goodness of fit, specifically designed for logistic regression models, frequently used in risk prediction tasks [66] to assess whether or not the observed event rates match the expected event rates in subgroups of the model population. Those subgroups are based on the deciles of the fitted risk values. HL is frequent among classical statistical studies [14,36,38,40,51].

In contrast with previous stances, the reviewed publications with *numerical outcomes* rely on residue-based predictive metrics. In this context, error metrics can be placed to assess how distant quantity predictions are from true observations, including the frequent mean absolute error (MAE) and root mean squared error (RMSE) metrics. In addition to MAE and RMSE, some studies also rely on the relative absolute error (RAE) and root relative squared error (RRSE) to obtain normalized views of the error [48]. In addition to statistical and ML regression methods, a few classification models with probabilistic outputs were also subjected to residue-based assessment prior to dichotomization, such as those in Danjuma [48]. Complementary to residue-based scores, the coefficient of determination, $R^{2}$ transduces the percentage of variation suffered by the outcome variable as explained by the independent variables, being a strong indicator of the goodness of fit. This metric is used in two of the reviewed studies [30,31].

The *validation* process should ensure the generalization ability of the postsurgical predictive models for new patients falling inside or outside the targeted population. In this context, cross-validation—specifically, leave-one-out cross-validation for small cohort studies—should be pursued. Understandably, preprocessing choices and hyperparameterization of the models should be conducted within the training partitions for a fair assessment. In this context, if cross-validation is further suggested for optimizing preprocessing and learning decisions, nested cross-validation—with inner and outer steps—should be considered [67]. Problems related to poor international applicability were highlighted by Garofallo et al. [68], Chin et al. [69], Formiga et al. [70] and Goh et al. [71]. The common conclusions pinpoint the need for further validation with foreign cohorts. As such, to guarantee the generalization ability for new populations, external validation should be further be pursued whenever possible, complementary to cross-validation (Figure 6) [5]. These studies also highlight that there are social and economic factors that should be included in the models to better support their generalization ability. Out of the surveyed works, only five out of twenty six do not refer to any validation means, perhaps due to data scarcity or the highly experimental character of contributions. For the remaining studies, the use of an independent validation set is more prevalent than the cross-validation setting [15,16,46,46].

## 5. Discussion

In previous sections, we journeyed through different approaches for postsurgical analysis of outcomes. The choice is inherently dependent on the available cohort data (Figure 2 and Figure 3) and desirable study outcomes (Figure 4), determining whether the focus should be placed on predictive models or outcome-conditional descriptive models (Figure 5) and which learning approach should be pursued.

*Classic statistical approaches* for postsurgical risk analysis (including point system and regression-based approaches) are inherently simple and interpretable. However, they are generally *unable* to do the following:

- Capture non-linear relationships within data;
- Translate risk scores into well-defined clinical decisions;
- Properly deal with the high-dimensional nature of clinical data;
- Identify local dependencies between variables;
- Be incrementally updated in the presence of new data;
- Tolerate arbitrarily-high levels of missing data;
- Explore the inherent temporal nature of clinical data.

Several extensions were proposed to minimize the impact of some of these limitations: (1) logistic regressions can be combined with decision trees to capture local regularities and guarantee a better ability to generalize in the presence of high-dimensional data; (2) conditional random fields can be applied to explicitly model dependencies between variables [72]; and, among others, (3) conditional logistic regressions can be pursued to take into account the population stratification and matching, particularly important given the diversity of demographic and cancer profiles as highlighted by the given taxonomy [73].

Moving from classical to *machine-learning* approaches brings unique opportunities of interest, namely a greater ability to capture non-linear and local relationships within data, while still placing principles to guarantee the ability of the models to generalize for new individuals. In accordance with Domingos [74] categorization, *symbolist approaches*—including associative classifiers and tree ensembles—are the most common option for postoperative care decisions. Decision trees are inherently simple, interpretable, provide a pattern-centric view of outcome-conditional associations, and can be enriched with statistical principles [75,76] to assess a decision’s significance. Still, they are susceptible to underfitting risks (losing relevant data that can support the discrimination of outcomes), as well as limitations when learning from numeric variables, a property associated with hypercubic decision boundaries. These limitations can be minimized by recurring to the ensembles of trees, based on bagging and bootstrapping principles, as shown by the promising performance of classifiers such as XGBoost [77] or CatBoost [78]. Despite their inherent merits, the interpretability is hampered, as well as efficiency (especially for those ensemble methods relying on stochastic gradients). Feature engineering is achieved by some of the ensemble methods to explore complex relationships among variables [79]. Although this possibility further degrades the interpretability of the models, mechanisms to show the relevance of each feature when placing decisions can be provided. To guarantee the ability to learn from temporal data, feature extraction is commonly applied, although associative classifiers based on discriminative temporal patterns are an increasingly common option toward longitudinal studies [80].

*Bayesian approaches* provide alternative principles for postsurgical risk analysis. They are inherently simple and the underlying graphical models can be extended to capture temporal dynamics—this is the case of hidden Markov models or dynamic Bayesian networks [81]. However, they are challenged by four major aspects. First, dependence on distribution assumptions. Second, the need to apply regularization principles to guarantee their ability to learn from high-dimensional data, specially for graphical models. Third, the need to place independence assumptions among groups of variables, even when considering Bayesian networks. Finally, as these approaches typically return one model per postsurgical outcome (class-conditional learning), there is an inherent difficulty on assessing how the values of certain variables (such as certain histopathological or surgical factors) affect the final decision.

*Analogizer approaches* constitute an additional possibility. Among them, lazy learning approaches, such as kNN, offer local decisions by focusing on individuals with similar demographic, physiologic and clinical profiles. In this context, they bypass the need to establish outcome-discriminative associations, thus handling with inherent simplicity the singularity of individual profiles, interventions, and present co-morbidities. On the downside, lazy learners suffer from three major challenges. First, they are dependent on adequate distance functions and neighborhood size (*k*). Second, the presence of mixtures of nominal–ordinal–numeric variables make difficult the assessment of the true distances between individuals. Third, the high-dimensionality of the available cohort data further challenges the assessment of similarities, even when the weight of variables is known and provided a priori [82]. Analogizer alternatives to lazy learning are kernel-based approaches, including support vector machines (SVMs), where dissimilarities are assessed to identify adequate decision boundaries. Despite their inherent merits, these approaches suffer from key limitations. First, SVMs are unable to properly handle categorical variables with medium-to-high cardinality. This is a severe drawback in the context of postsurgical risk analysis since such variables represent a good portion of commonly available variables from the target cohort studies [83]. Second, they are dependent on the selection of adequate kernels to learn non-linear decision boundaries. Third, kernel-based models generally lack interpretability. Finally, they are susceptible toward overfitting risk in the absence of proper regularization principles. A limitation transversal to analogizers is the presence of missing postsurgical clinical data, which generally needs to be imputed, creating biases.

Among the learning paradigms, *connectionist approaches* for clinical data analysis have seen a resurgence in the last decades with the advent of deep learning, and hold particular properties of interest for the specific aim of analyzing postsurgical outcomes [7]. First, they are inherently able to learn from high-dimensional data, possibly combining non-iid and temporal variables. Second, they are inherently prepared to capture complex non-linear relationships without the need to establish assumptions regarding the nature of the regularities underlying data (Figure 3). Despite their inherent merits, the efficacy of connectionist approaches depends on the adequacy of the underlying architecture and on proper hyperparameter choices. Fixing architectural decisions (e.g., layering, or activation) is generally a computationally complex step. In addition, neural networks generally (1) lack interpretability, (2) are unable to provide statistical guarantees on the adequacy of decisions, and (3) depend on the availability of a considerably large cohort of studies to guarantee learning convergence. With the aim of addressing these challenges, recent contributions in the field offer the possibility to (1) extract visual representations on the underlying network patterning [84], (2) provide a Bayesian frame to neural network learning for statistical assessments [85], and (3) have pairwise learning principles for data augmentation [86].

### 5.1. On the Interpretability of Predictive Models

Among the diversity of introduced quality aspects (Figure 6), the interpretability of machine-learning predictors is a major demotivator for their clinical translation. There is already a diverse yet disperse set of contributions aimed at fostering the interpretability and explainability of ML models [87,88,89]. White-box models are inherently simple and provide some type of clear justification associated with the decision while also providing insight into the internal structure of the model. Linear regression models and decision trees fall into this category, either for their simple mathematical foundations or their intuitive visual representation [89]. Black-box models bring new dimensions to the concept of interpretability, often coming in the form of non-representative justifications of a decision [87]. The peak of black-box models is consensually found in connectionist approaches. However, strategies have already been developed to mitigate this downside of otherwise very powerful predictors. Layer-wise relevance propagation (LRP) is a technique that offers explainability and is able to scale to complex deep neural networks, operating by propagating the prediction backwards in the network, aiming to explain the factors leading to a decision [90]. Analogous mechanisms are used by complex associative models through feature importance, as seen in XGBoost [77] or CatBoost [78]. Surrogate models further complement the tool set for interpretability extraction and enhancement. Rationalizers propose a novel approach for incorporating rationale generation as an integral part of the overall learning process [91]. From this perspective, the rationales are simply sub-sets of the inputs able to yield the same prediction, therefore qualifying as an explanation. Local interpretable model-agnostic explanations (LIME) is another technique that is model agnostic, learning the behavior of a given model by perturbing the input and watching how the predictions change [92]. The idea behind LIME is that a model may be complex to explain globally; however, it is easier to approximate the model around the proximity of a particular instance. This principle fosters interpretability and further supports validation in clinical contexts by allowing healthcare professionals to undergo model perturbations in accordance with the surgical, histopathological, molecular and demographic profile of the individuals under assessment.

### 5.2. A final note on quantitative assessments

Motivated by the need to account for the diversity of the introduced taxonomic dimensions, a cohort study was conducted with 847 patients who had undertaken surgery for cancer treatment at the IPO-Porto cancer center, Porto, Portugal. Different areas of surgical oncology (Figure 2) were considered and more than 120 clinical variables were collected, comprising most of the dimensions introduced in Figure 3. Considering this recent initiative, we employed an extensive set of ML models (Figure 5) to predict four outcomes of interest: the existence of postoperative complications, the complications’ severity, the ICU length of stay, and 1-year death after surgery [93].

The results (Figures S1–S4 in Supplementary Material), after cross-validation, showed variations on the performance of the surveyed predictors in accordance with the given input variables and selected outcome. We were able to verify in practice many of the aspects described in this review, such as the suitability of associative models, such as random forests and XGBoost, for clinical contexts as previously highlighted by Domingos [74].

This preliminary study stresses the relevance of carefully considering the introduced variables along the dimensions of the proposed taxonomy to aid the design of cohort studies, assist the development or selection of predictive approaches, and properly assess the accuracy, generalization, updatability and interpretability of the proposed prognostication tools to guarantee their proper clinical translation.

## 6. Conclusions

This paper proposes a structured view of the problem of predicting postoperative complications within the oncological domain, surveying the main contributions in the field. To this end, we first established a taxonomy to (1) assess the opportunities and challenges of existing cohort studies with regards to their scope, postsurgical outcome and data collection, and (2) guide the development and evaluation of learning approaches to study and predict postoperative complications. Contextualized by this taxonomy, the work then provided a comprehensive overview of classical and machine learning approaches for postsurgical risk analysis. A qualitative comparison was offered by taking into consideration the available cohorts per study, as well as the targeted outcomes, either associated with morbidity aspects (such as the occurrence, nature or severity of postsurgical complications and hospitalization needs) or mortality concerns.

This study shows that the area of postsurgical risk analysis is still in its infancy, as the existing approaches often neglect important demographic, biophysiological and clinical variables, particularly those pertaining to the nature of interventions, postsurgical care and recovery. In addition, the inherent heterogeneous, temporal and structurally sparse nature of pre- and postoperative data is generally disregarded. As more and more high-quality data from multi-hospitals become available, novel integrative learning approaches able to tackle these challenges are expected, particularly driven by the need to guarantee the generalization ability, sensitivity, updatability and statistical significance of the predictive models. From our point of view, this is just possible through multidisciplinary collaborations between health professionals and data scientists.

The present study is being conducted in the context of the IPOscore project, a project that aims to consider the aforementioned findings for postsurgical risk analysis as well as address some of the shortcomings of available cohort studies through a comprehensive monitoring of demographic, biologic, histopathologic and clinical aspects from a population of cancer patients subjected to surgical interventions.

## Figures and Tables

**Table 1:** Compilation of traditional statistical studies in postoperative prognostics according to the major taxonomic dimensions (literature references in chronological order).

Study	Surgical Cohort	Model	Data Type	Data Size	Validation	Outcome
Saklad [10]	General	N/A	N/A	N/A	N/A	Morbidity, Mortality
Knaus et al. [30]	General	LR	Clinical	5815	Yes	In-Hospital Death
Charlson et al. [31]	General	WI	Clinical	559	Yes	1-Year Mortality
Copeland et al. [13]	General	LR	Clinical	1372	N/A	Morbidity, Mortality
Marcantonio et al. [32]	Noncardiac	LR	Clinical	876	Yes	Postoperative Delirium
Whiteley et al. [33]	General	LR	Clinical	10,000	Yes	Morbidity, Mortality
Roques et al. [34]	Cardiac	LR	Clinical	19,030	N/A	Mortality
Dupuis et al. [35]	Cardiac	LR	Clinical	3548	N/A	Morbidity, Mortality
Arozullah et al. [36]	Noncardiac	LR	Clinical	160,805	Yes	Postoperative Pneumonia
Sutton et al. [37]	General	LR	Clinical	3144	Yes	Morbidity
Donati et al. [38]	Cardiac	LR	Clinical	1936	Yes	Mortality
Gawande et al. [39]	General	PS	Clinical	303	Yes	Morbidity, Mortality
Canet et al. [40]	General	LR	Clinical	2464	Yes	Pulmonary Complications
Gupta et al. [41]	General	LR	Clinical, demographic	211,410	Yes	Cardiac Complications
Vaid et al. [42]	General	LR	Clinical, demographic	202,741	Yes	Mortality
Bilimoria et al. [14]	General	LR	Clinical, demographic	1,414,006	Yes	Morbidity, Mortality

LR = Logistic Regression; PS = Point System; WI = Weighted Index; N/A = Not Available.

**Table 2:** Compilation of machine-learning studies in postoperative prognostics, according to the major taxonomic dimensions (literature references in chronological order).

Study	Surgical Cohort	Model	Data Type	Data Size	Validation	Outcome
Khan et al. [45]	Breast	Fuzzy DT	Clinical, Biological	162,500	Yes	5-year mortality
Chang et al. [46]	Oral	NN, Fuzzy NN, SVM, LR	Clinical, histopathological, genetic	31	Yes	3-year mortality
Zięba et al. [47]	Lung	Boosted SVM	Clinical, histopathological	1200	N/A	1-year survival
Danjuma [48]	Lung	MLP, DT, NB	Clinical	470	Yes	1-year mortality
Parmar et al. [49]	Head and neck	NB, RF, NN	Radiomics	101	Yes	3-year mortality
Wang et al. [50]	Bladder	NB, SVM, kNN, NN	Clinical, histopathological	117	Yes	5-year mortality
Thottakkara et al. [51]	Major surgery	LR, GAM, SVM, NB	Demographic, socioeconomic, clinical, laboratory	50,318	Yes	Postoperative sepsis and kidney injury
Soguero-Ruiz et al. [52]	Colorectal	SVM	Physiological, clinical	402	Yes	Anastomosis leakage
Kim et al. [53]	Oral	NN	Clinical, histopathological	255	Yes	5-year mortality
Parikh et al. [54]	General oncology	LR, GB, RF	Demographic, laboratory, comorbidities	26,525	Yes	180-day and 500-day mortality

NN = Neural Network; DT = Decision Tree; LR = Logistic Regression; GB = Gradient Boosting; RF = Random Forest; NB = Naive Bayes; GAM = Generalized Additive Model; SVM = Support Vector Machine; kNN = k-Nearest Neighbors; MLP = Multilayer Perceptron.

## Data Availability

The data used for gathering the results in supplementary material are not publicly available due to confidentiality reasons.

## Supplementary Materials

The following are available online at https://www.mdpi.com/article/10.3390/cancers13133217/s1, Figure S1: Best model results for postoperative complications prediction (RF = Random Forest; MLP = Multi-Layer Perceptron; SVM = Support Vector Machine; CBC = CatBoost Classifier; LR = Logistic Regression); Figure S2: Best model results for complications’ severity prediction (RF = Random Forest; CBC = CatBoost Classifier; DT = Decision Tree; SVM = Support Vector Machine; XGB = XGBoost); Figure S3: Best model results for ICU length of stay prediction (Ridge = Ridge Regression; Linear = Linear Regression; PLS = Partial Least Squares; MLPR = Multi-Layer Perceptron Regressor; RF = Random Forest); Figure S4: Best model results for 1-year death prediction (RF = Random Forest; CBC = CatBoost Classifier; XGB = XGBoost; SVM = Support Vector Machine; NB = Naive Bayes).

## Abbreviations

ASA-PS	American Society of Anesthesiologists for the classification of Physical Status
ACS	American College of Surgeons
ANFIS	Adaptive Neuro-Fuzzy Inference System
ANN	Artificial Neural Network
CARES	Combined Assessment of Risk Encountered in Surgery
AUC	Area Under the Curve
DT	Decision Tree
ELM	Extreme Learning Machines
FP	False Positive
FN	False Negative
HL	Hosmer–Lemeshow Test
IPO	Instituto Português de Oncologia
GAM	Generalized Additive Model
GB	Gradient Boosting
kNN	k-Nearest Neighbors
LR	Logistic Regression
MAE	Mean Absolute Error
ML	Machine Learning
MLP	Multi-Layer Perceptron
NB	Naive Bayes
NN	Neural Network
NSQIP	National Surgical Quality Improvement Program
POSSUM	Physiological and Operative Severity Score for enumeration of Mortality and Morbidity
PS	Point System
TP	True Positive
TN	True Negative
RAE	Relative Absolute Error
RELM	Regularized Extreme Learning Machines
RF	Random Forest
ROC	Receiver Operating Curve
RMSE	Root Mean Squared Error
RRSE	Root Relative Squared Error
SVM	Support Vector Machine
WI	Weighted Index
XGBoost	Extreme Gradient Boosting
