# Supplementary material for: Predicting Postoperative Complications in Cancer Patients: A Survey Bridging Classical and Machine Learning Contributions to Postsurgical Risk Analysis
Source: Cancers (Basel). 2021 Jun 28;13(13):3217. doi: 10.3390/cancers13133217 (PMC8269422; doi:10.3390/cancers13133217)
Supplement: Supplementary file 1 [file cancers-13-03217-s001.zip › cancers-1219622-supplementary.pdf]

cancers

Supplementary Materials

Predicting Postoperative Complications in Cancer Patients: A Survey Bridging Classical and Machine Learning Contributions to Postsurgical Risk Analysis

Daniel M. Gonçalves, Rui Henriques, Rafael S. Costa

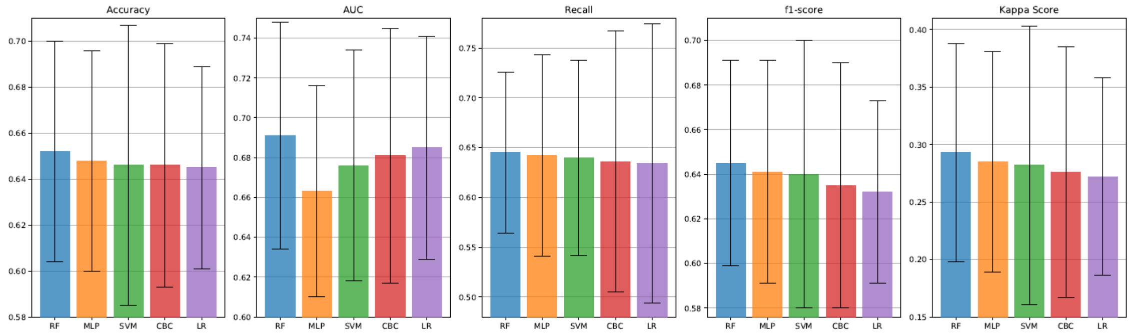

Figure S1. Best model results for postoperative complications prediction (RF=Random Forest; MLP=Multi-Layer Perceptron; SVM=Support Vector Machine; CBC=CatBoost Classifier; LR=Logistic Regression.)

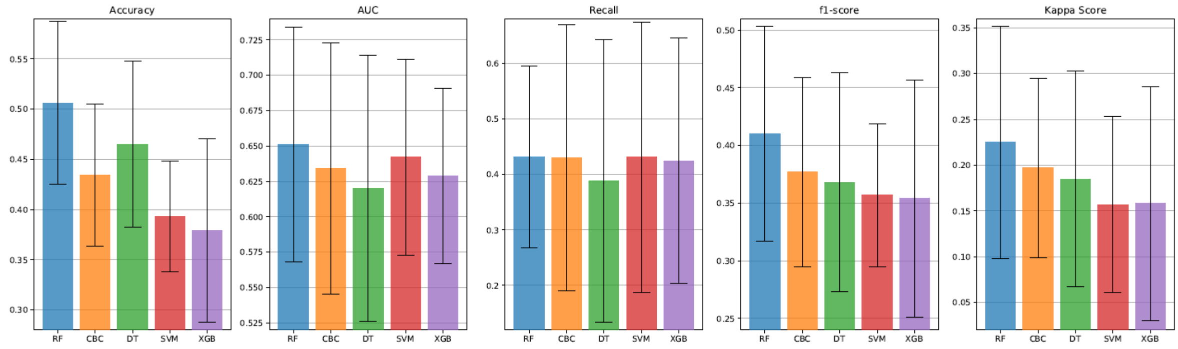

Figure S2. Best model results for complications' severity prediction (RF=Random Forest; CBC=CatBoost Classifier; DT=Decision Tree; SVM=Support Vector Machine; XGB=XGBoost.)

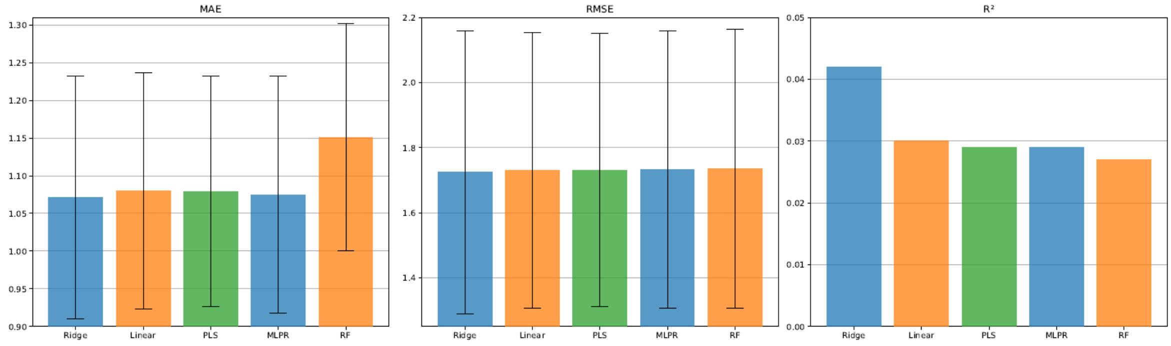

Figure S3. Best model results for ICU length of stay prediction (Ridge=Ridge Regression; Linear=Linear Regression; PLS=Partial Least Squares; MLPR=Multi-Layer Perceptron Regressor; RF=Random Forest.)

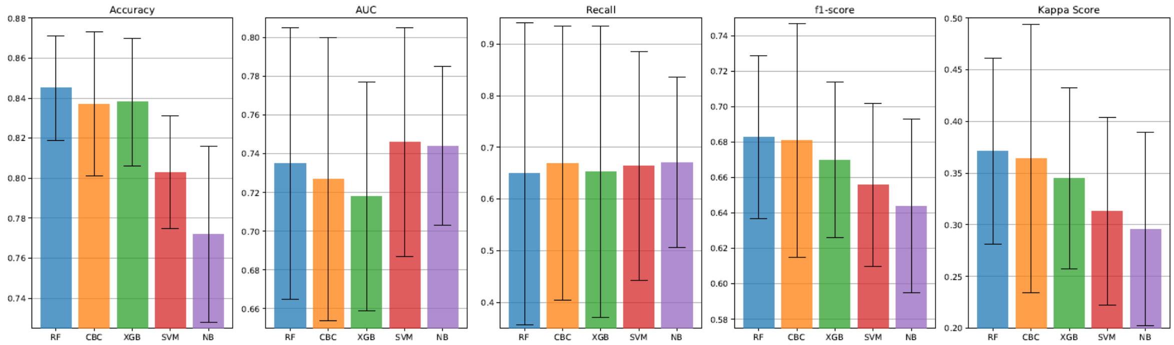

Figure S4. Best model results for 1-year death prediction (RF=Random Forest; CBC=CatBoost Classifier; XGB=XGBoost; SVM=Support Vector Machine; NB=Naive Bayes.)
